# *Cuscuta reflexa* Roxb. Expedites the Healing Process in Contact Frostbite

**DOI:** 10.1155/2020/4327651

**Published:** 2020-10-02

**Authors:** Waseem Hassan, Manal Ali Buabeid, Umme Kalsoom, Sahar Bakht, Imran Akhtar, Furqan Iqbal, El-Shaimaa A. Arafa

**Affiliations:** ^1^Department of Pharmacy, COMSATS University Islamabad, Lahore Campus, Lahore 54000, Pakistan; ^2^College of Pharmacy and Health Sciences, Ajman University, Ajman 346, UAE; ^3^Department of Pharmacy, The Islamia University of Bahawalpur, Pakistan; ^4^Department of Pharmaceutics, Bahauddin Zakariya University, Multan, Pakistan; ^5^Department of Pharmacology and Toxicology, Faculty of Pharmacy, Beni-Suef University, Beni-Suef, 62514, Egypt

## Abstract

Frostbite is caused due to extreme vulnerability to cold, resulting in damage of deeper and superficial tissues alike. In this study, we report the anti-inflammatory and wound-healing properties of aqueous methanolic extract of *Cuscuta reflexa* (*Cs.Cr*) against contact frostbite. Thirty rats were divided into five groups including three treatment groups with increasing doses of *Cs.Cr*, a standard drug group receiving acetylsalicylic acid (ASA), and a metal bar-induced frostbite group. Frostbite injury was induced by a 3 × 3.5 cm metal bar frozen up to -79°C on shaved skin for continuous 3 minutes. Wounded area percentages were recorded to measure the healing rate in response to *Cs.Cr* administration. Haematological parameters and malondialdehyde content were also noted. On treatment with *Cs.Cr*, the healing rate is drastically increased and lipid peroxidation product malondialdehyde was decreased in a dose-dependent manner. Results were compared with frostbite and ASA (standard drug group). These results indicate that *Cs.Cr* possesses excellent wound-healing properties against frostbite injury and can prove to be a prospective compound in such conditions.

## 1. Introduction

Frostbite is an injury caused by freezing of the skin and underlying tissues commonly caused by exposure to cold-weather conditions and contact with ice, freezing metal, or cold liquids. It represents a continuum of tissue injuries, ranging from superficial insult to deep tissue damage [1]. Although a comprehensive epidemiological study is yet to be surfaced, it is estimated that 366 out of 1000 mountaineers suffer frostbite per year [2]. It is assessed that men in the age bracket of 30-49 are most at risk of frostbite due to greater chances of exposure. Expectedly, frostbite has been reported to affect military personnel [3] and mountaineers [4] followed by the general population [5]. Frostbite usually occurs at the exposed area where temperature is below the freezing point, i.e., -2°C (28°F). Its severity depends upon the exposure time [6]. Frostbite injury causes loss of productivity, pain, and economic burden and has potential of permanent disability [3]. The pathogenesis of frostbite is not very clear, but according to reported studies, it includes direct cold damage to tissues and injury from ice crystal formation which causes protein disruption [7], arterial vasoconstriction, thrombus formation, and ischemia [8]. The suitable treatment of frostbite is chosen depending upon its severity and duration [9]. The clinical treatments available for frostbite are anticoagulation therapy [10], vasodilators [11, 12], thrombolytic therapy, sympathectomy [9], hyperbaric oxygen [13, 14], and surgical treatment.

Currently, there are fewer specific therapies to frostbite injuries. Aloe vera is the only plant which has manifested reduction in tissue loss and has achieved a certain degree of success in the treatment of frostbite. According to WHO estimates, about three-quarters of the world's population use herbs for different illnesses. *Cuscuta reflexa* Roxb. from the Cuscutaceae family, commonly known as “Akashabela,” “Amarabela,” or “Loot,” is a perennial herb. The genera *Cuscuta* includes 170 parasitic species [15]. This parasitic plant species is a rootless, leafless twined sprawling thin vine that grows over a host plant. It has a heterotrophic mode of nutrition due to lack of chlorophyll and hence is completely dependent on the host plant for survival [16]. The utilization of the entire plant of *Custuca reflexa* is linked with the treatment of different illnesses. For instance, it is devoured as a therapeutic option for cerebral pain, tingling [17], headache, skin rashes, amnesia, epilepsy, cough, and fever [18]. The aqueous ethanolic extract of *Cuscuta reflexa* has been studied for its constituents and found to contain alkaloids, tannins, flavonoids, and phenolic compounds [19–21]. The plant has scientifically been investigated for various pharmacological activities like anxiolytic activity [22], hair growth potential [18, 23, 24], antitumor activity [25], and antibacterial activity [26, 27]. The crude extract has also been evaluated for antiepileptic and *α*-glucosidase inhibition [28]. Moreover, this plant inhibits the erythrocyte damage due to hypotonicity which is anti-inflammatory effect [29]. Additionally, the plant has been quarantined for antitumor and antiproliferative activities using different animal models [25].

Despite its traditional applications in skin diseases, there is no data available in scientific literature depicting the relationship of anti-inflammatory and wound-healing property of this plant against any cold injury [17, 30]. Encouraging anti-inflammatory activities along with its effectiveness in skin disorders motivated us to evaluate its activities in frostbite.

The aim of the study is to evaluate the healing role of *Cs.Cr* in frostbite that may prevent tissue loss and subsequent amputation.

## 2. Materials and Methods

### 2.1. Plant Material

A whole fresh plant of *Cuscuta reflexa* was collected from the vicinity of Bahawalpur City (Southern Punjab, Pakistan). It was identified from an authentic botanist, and a voucher number was issued, i.e., CR-WP-05-10-006. The plant sample was preserved for future reference in the herbarium of the Faculty of Pharmacy and Alternative Medicine, the Islamia University of Bahawalpur, Pakistan. The whole plant part of *Cuscuta reflexa* was dried under shade to weed out for any superfluous material.

### 2.2. Preparation of Crude Extract

The dried plant was then coarsely powdered in a blender. Then, it was macerated with 70% aqueous methanol for three days. The macerate was passed through muslin cloth followed by filter paper. The filtrate was evaporated in a rotary evaporator (Heidolph Laborota 4000 efficient, Germany). Finally, crude extract was collected in a China dish in the form of thick viscous liquid. It was further dried in a hot air oven at 40°C. The crude extract was weighed and stored in a freezer for phytochemical and pharmacological analysis.

### 2.3. Chemicals

The chemicals utilized during research work were acquired from different sources. Acetylsalicylic acid (ASA) was purchased from the local market; ketamine from chemical works of Gedeon Richter Ltd., Budapest, Hungary; and xylazine from Prix Pharmaceutica, Lahore. Hair removing cream Veet was purchased from the local market. All the doses of *Cs.Cr* crude extract (200, 400, and 800 mg/kg) and acetylsalicylic acid (50 mg/kg) were prepared in normal saline and administered as 4 mL/kg P.O.

### 2.4. Experimental Animals

Wistar albino rats weighing 130-190 g were kept at control temperature (26 ± 2°C) in a 12 hr light and dark cycle. They were provided with standard rodent feed and water *ad libitum.* The study protocols were approved by the institutional committee, i.e., PREC (Pharmacy Research Ethics Committee) of the Department of Pharmacy, Faculty of Pharmacy and Alternative Medicine, the Islamia University of Bahawalpur.

### 2.5. Induction of Frostbite

**Figure 1:**
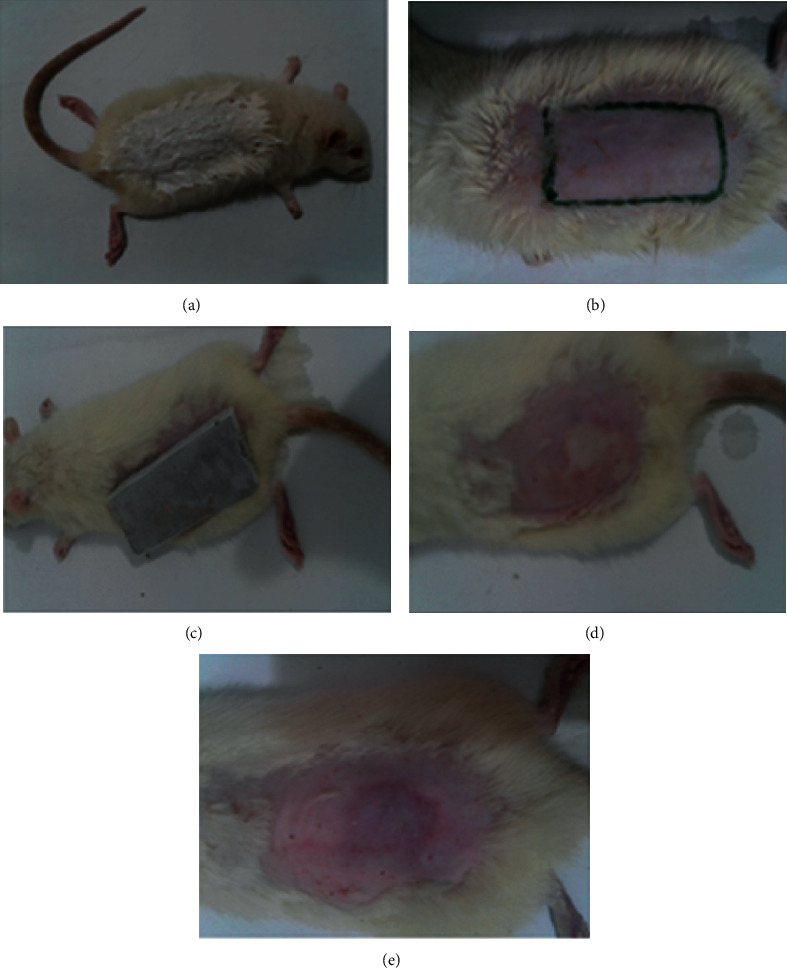
Process of induction of frostbite in Wistar rats. (a) Hair on the dorsal area is removed. (b) Area to be used is marked on the animal's back. (c) Frozen metal bar is placed on the prepared skin. (d) Skin is shown immediately after thawing. (e) Skin is shown after 3 h of thawing.

We have followed the Chigunadze et al. model to induce frostbite [31] with minor modifications. Animals were anesthetized with ketamine and xylazine (10 : 1), and hair was removed with hair removal cream (Veet cream). It was applied for 3 minutes, and then, cream was removed with dried and wet cotton swabs, respectively. Simulated contact frostbite was produced with the help of metal weighing 3.5 × 3 cm which was precooled up to -73°C in a freezer. It was then made to contact the shaved area of the rat's skin for 3 minutes (Figure 1). After induction of injury, animals were returned to their cages for completion of the thawing process at room temperature. Standard and test drug treatment was started after 24 h.

### 2.6. Biochemical Assay

A blood sample was collected from the animals' eye using nonheparinized capillary tubes. The determined parameters include platelet count, total leukocyte count, hemoglobin, WBCs, and RBCs, although only platelet count and WBCs showed significant changes.

### 2.7. Determination of Lipid Peroxidation

Malondialdehyde, as a marker for lipid peroxidation, was determined in serum by the double heating method of Draper and Hadley with some modifications. The principle of the method is based on spectrophotometric measurement of the color produced during the reaction of TBA with malondialdehyde. For this purpose, 2.5 mL of 100 g/L trichloroacetic acid solution was added into 0.5 mL serum in a centrifuge tube and placed in a boiling water bath for 15 min. After cooling under tap water, the supernatant was transferred into a test tube containing 1 mL of 6.7 g/L TBA solution and placed again in a boiling water bath for 15 min. The solution was then cooled under tap water, and its absorbance was measured spectrophotometrically at 532 nm. The concentration of malondialdehyde was calculated using the following equation: malondialdehyde (nmol/mL) = [(Absorbance of sample/Absorbance of standard) × 100].

### 2.8. Wound Area Analysis

Photographs were taken by a digital camera on alternative days, and images were then analyzed by ImageJ software (National Institutes of Health, Bethesda, MD) [32]. The wound area was calculated as Wound Area = Original wound area–wound area on day *X*/original wound area.

### 2.9. Analysis of Percentage Wound Area Recovered

The wound area that recovered was measured from the photographs by using ImageJ software (National Institutes of Health, Bethesda, MD). Then, the percentage of the wound area was calculated as %Wound Area Recovered = (Original wound area–wound area on day *X*/original wound area) × 100.

### 2.10. Statistical Analysis

The values were expressed as the mean ± SEM. Statistical analysis was performed with two-way ANOVA followed by Bonferroni's test. Results were considered nonsignificant (∗) if *P* < 0.05, significant (∗∗) if *P* < 0.01, and highly significant (∗∗∗) if *P* < 0.001. The data was compiled and statistically analyzed by using GraphPad Prism version 5.

## 3. Results

### 3.1. *Cuscuta reflexa* Crude Extract Decreases the Area and Percentage of Wound

**Figure 2:**
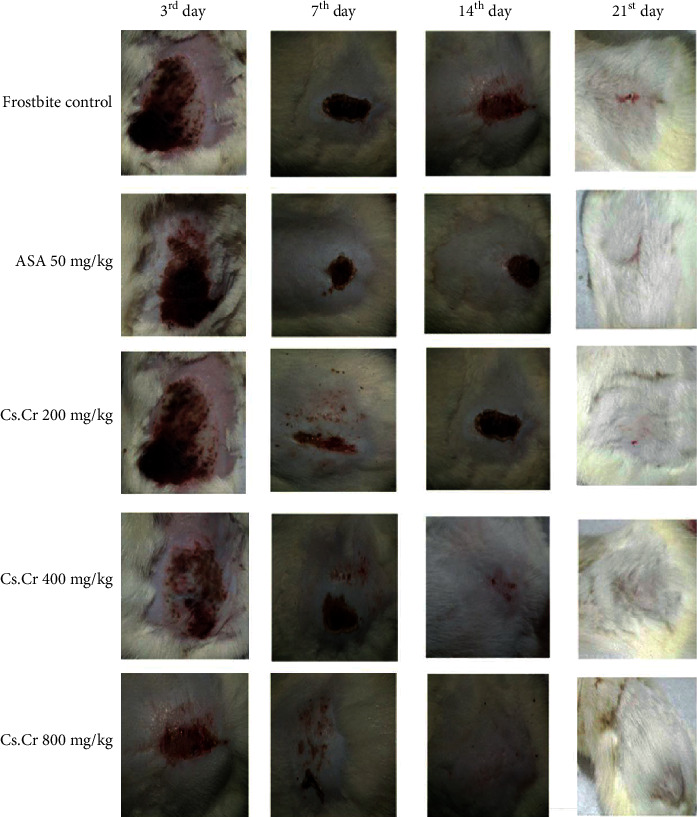
Frostbite wound progression/healing during the phase of the study. Animals were divided into groups including frostbite control and three Cs.Cr-administered groups. Photographs were taken after the 3^rd^, 7^th^, 14^th^, and 21^st^ days after frostbite was induced with a metal bar. The figure shows the representative photographs from respective groups.

**Figure 3:**
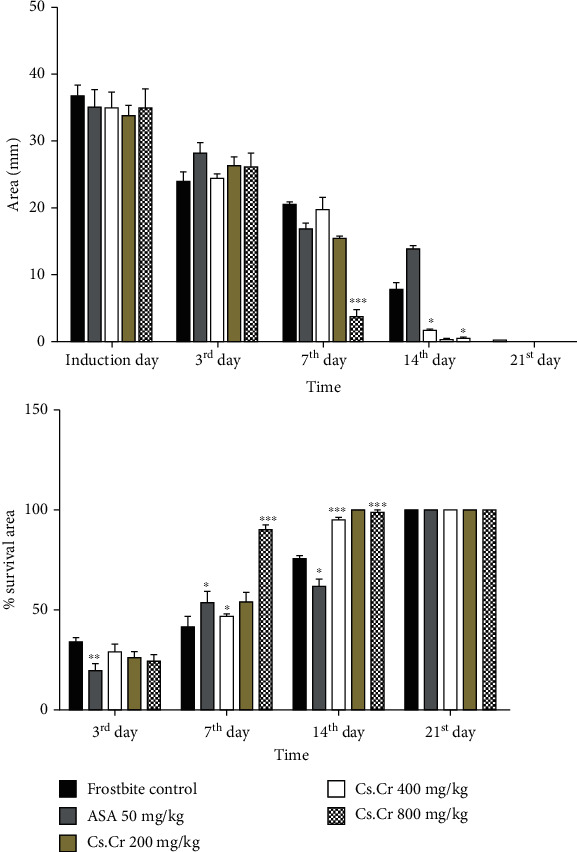
Graphical representation of the frostbite wound-healing area and percentage survival area. (a) Wound area reduction in mm; (b) percentage survival area during the phase of the study. The graph shows the manifestation of values (mm) and percentages on frostbite induction on the 3^rd^, 7^th^, 14^th^, and 21^st^ days. The values are expressed as the mean ± SEM of six animals in a group. Statistical analysis is performed with two-way ANOVA followed by Bonferroni's test. All the groups are compared with the frostbite control group. Results are nonsignificant (∗) if *P* < 0.05, significant (∗∗) if *P* < 0.01, and highly significant (∗∗∗) if *P* < 0.001.

The Cs.Cr remarkably reduced the wound area both dose- and time-dependently as shown in Figures 2 and 3. The frostbite-induced group showed signs of relative wound healing at the end of the study (21^st^ day), while similar results were obtained in the Cs.Cr (800 mg/kg)-administered group after the 7^th^ day sparing significant and permanent tissue losses (Figures 2 and 3).

### 3.2. Effect of Crude Extract of *Cuscuta reflexa* on Complete Blood Count (CBC)

**Figure 4:**
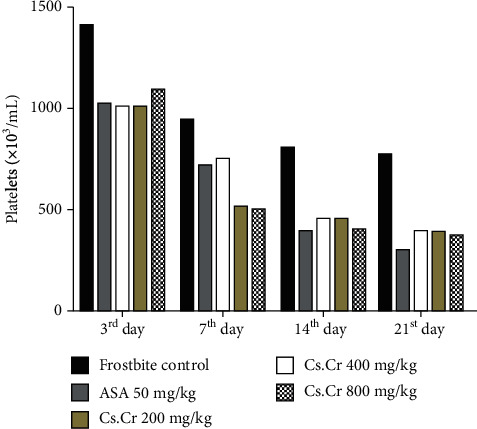
Graph showing the effect of *Cuscuta reflexa* extract on platelet count. Statistical analysis is performed with two-way ANOVA followed by Bonferroni's test. All the groups are compared with the frostbite control group. Results are nonsignificant (∗) if *P* < 0.05, significant (∗∗) if *P* < 0.01, and highly significant (∗∗∗) if *P* < 0.001.

CBC of the experimental animals is performed on respective sampling days. Expectedly, no other parameter shows the variation except the platelet count. Platelet count increases immediately after induction of injury. This marked increase is countered by the *Cs.Cr* as shown in Figure 4.

### 3.3. Effect of *Cuscuta reflexa* Extract on Malondialdehyde

Flavonoids have antioxidant activity derived from plants [33]. Frostbite is virtually due to oxidative stress, and this ongoing process is generally measured by secondary products such as lipid peroxidation [34]. Malondialdehyde is a potent indicator of the lipid peroxidation level [35, 36].

**Figure 5:**
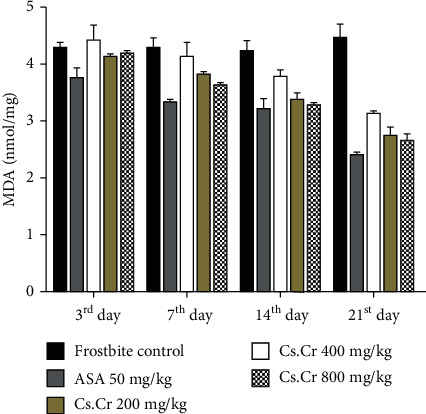
The graphs showing the MDA (nmol/mg) content of frostbite on respective study days. Comparison is established between the frostbite control group and the treatment groups. Statistical analysis is performed with two-way ANOVA followed by Bonferroni's test. All the groups are compared with the frostbite control group. Results are nonsignificant (∗) if *P* < 0.05, significant (∗∗) if *P* < 0.01, and highly significant (∗∗∗) if *P* < 0.001.

The frostbite control group shows an increase in lipid peroxidation content till the 21^st^ day. Treatment groups and standard groups were compared with the frostbite control group. The treatment group with a dose 800 mg/kg showed a marked decrease in malondialdehyde content, i.e., 1.68 nmol/mg on the 3^rd^ day, and it is significant (*P* < 0.01), and there is a highly significant (*P* < 0.001) decrease on the 14^th^ day, i.e., 1.33 nmol/mg. Cs.Cr given in doses 200, 400, and 800 mg/kg shows a dose-dependent activity as shown in Figure 5 and Table 1.

## 4. Discussion

Frostbite is a common occurrence in colder zones of the globe experienced by the local population, sportsmen, and tourists. It usually occurs when skin temperature drops below -0.5°C [37]. With the rise in snowstorms, avalanches, growing tourism, climate change, and increase in global sporting activities, the frostbite events are naturally on the rise. Amputation of limbs and sepsis are the major frostbite complications [38] that demand the attention of the scientific community. Our goal of the study was to search and analyze the effects of *Cs.Cr* on an induced frostbite model [32]. The metal bars were utilized for freezing dorsal tissue and observing wound-healing progression through granulation and reepithelialisation. This model allows quantification of the affected skin surface area, histology, healing rate, and skin loss [32]. Previously reported models are good at explaining the wound-healing process after frostbite injury, but they are complex because of radioisotopes and blood vessels overlapping [32].

Our study results show that necrosis of tissues can be induced by a frozen metal bar after it makes contact with the skin. Eschar appears after 2 to 3 days (shown in Figure 2). Visual inspection can be done, and therapy administered can give visible effects. Likewise, the treatment effects of *Cs.Cr* were palpable during the course of the study. *Cs.Cr* dose- and time-dependently reduced the area and percentage of induced frostbite wound at the dose of 200 mg/kg, 400 mg/kg, and 800 mg/kg. Positive changes in the healing process were evident even in the first seven days at the dose of 200 mg/kg of *Cs.Cr*, possibly preventing the early tissue loss and damage to the deeper organs. The positive changes continued till the 21^st^ day. This reflects the anti-inflammatory and early wound-healing properties of Cs.Cr in frostbite, which is particularly important in saving the tissue damages that usually occur within the first few hours of injury. ASA was used as a positive control drug for its well-known wound-healing and anti-inflammatory activities.

Increased concentration of malondialdehyde is an indication of lipid peroxidation [39]. Malondialdehyde is the oxidation product of fatty acids. The malondialdehyde level in rats after the exposure to cold in frostbite is due to the generation of free radicals. Our study indicates that the increased level of malondialdehyde is controlled by the administration of *Cs.Cr* effectively. Therefore, it can be concluded that it can scavenge free radicals and thus can reduce oxidative stress.

The phytochemical constituents that are present in medicinal plants possess medicinal and physiologic properties [40]. It was revealed that *Cs.Cr* was particularly rich in flavonoids, quinones, and alkaloids (Table 1). Our results are in agreement with the previous findings that suggest the anti-inflammatory [20] and wound-healing properties of flavonoids [41]. Furthermore, the phytochemical screening of *Cs.Cr* is already reported and connected with its wound-healing properties [42].

## 5. Conclusion

Conclusively, *Cs.Cr* is significantly effective in frostbite through the reduction of inflammation, although further studies should be performed to identify the molecular mechanism of these biological activities.

## Figures and Tables

**Table 1:** The results of phytochemical evaluation of crude extract of *Cs.Cr* (+) indicate the presence of constituents.

Phytochemical constituents	Cs.Cr
Tannins	+
Saponins	+
Flavonoids	+++
Phenols	+
Quinones	++
Alkaloids	++
Proteins	+

## Data Availability

The data used to support the findings of this study are available from the corresponding author upon request.

## Supplementary Materials

Supplementary Materials Graphical abstract.
