# Supplementary figures and images for: Cuscuta reflexa Roxb. Expedites the Healing Process in Contact Frostbite
Source: Biomed Res Int. 2020 Oct 2;2020:4327651. doi: 10.1155/2020/4327651 (PMC7556107; doi:10.1155/2020/4327651)

**Graphical abstract**
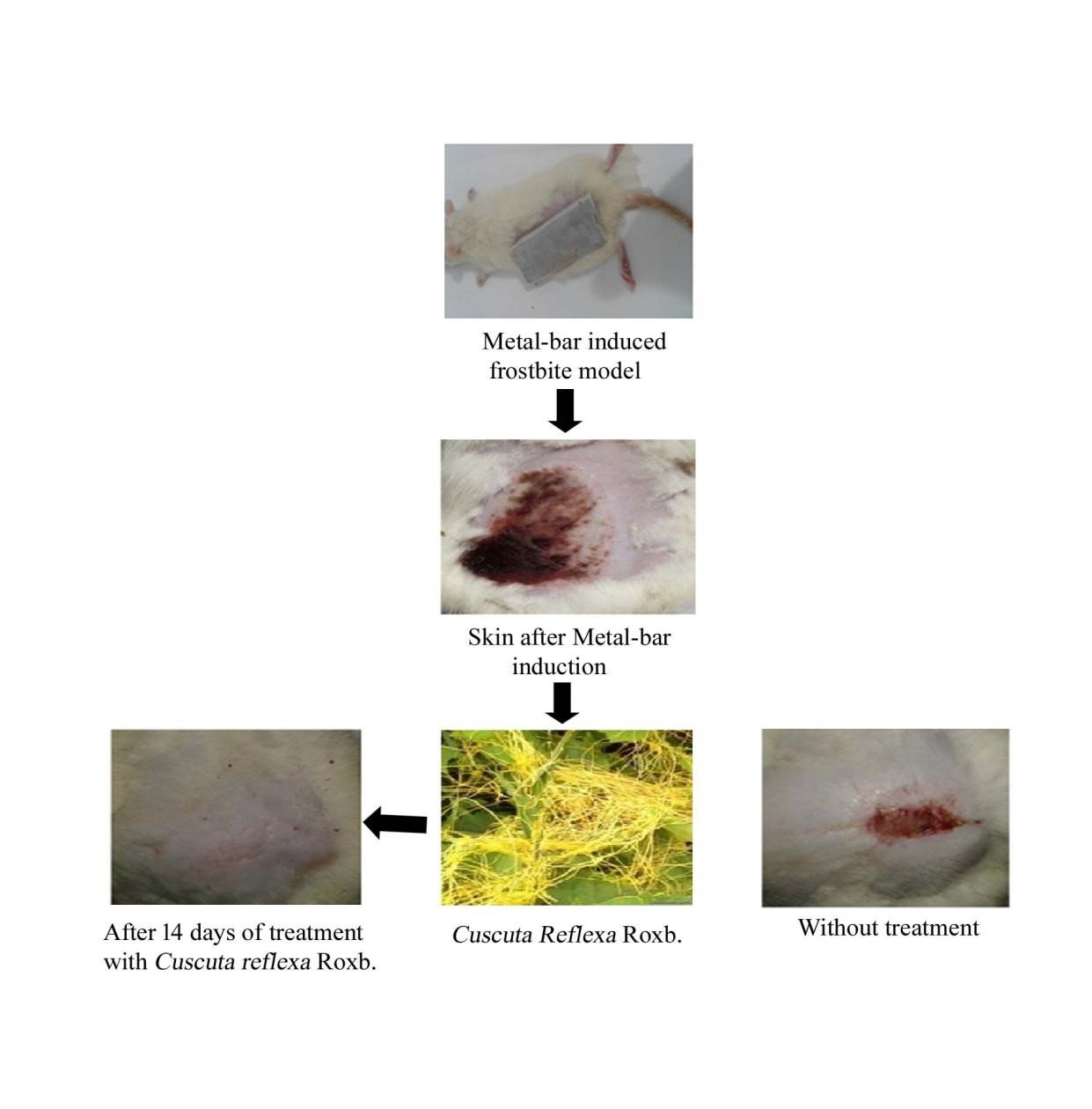

Supplement: Supplementary Materials — Graphical abstract. [file 4327651.f1.docx]
